# Supplementary material for: [68Ga]Ga-DOTAGA-Glu(FAPi)2 Shows Enhanced Tumor Uptake and Theranostic Potential in Preclinical PET Imaging
Source: Diagnostics (Basel). 2024 Sep 13;14(18):2024. doi: 10.3390/diagnostics14182024 (PMC11431137; doi:10.3390/diagnostics14182024)
Supplement: Supplementary file 1 [file diagnostics-14-02024-s001.zip › diagnostics-3159820-supplementary.pdf]

diagnostics

MDPI

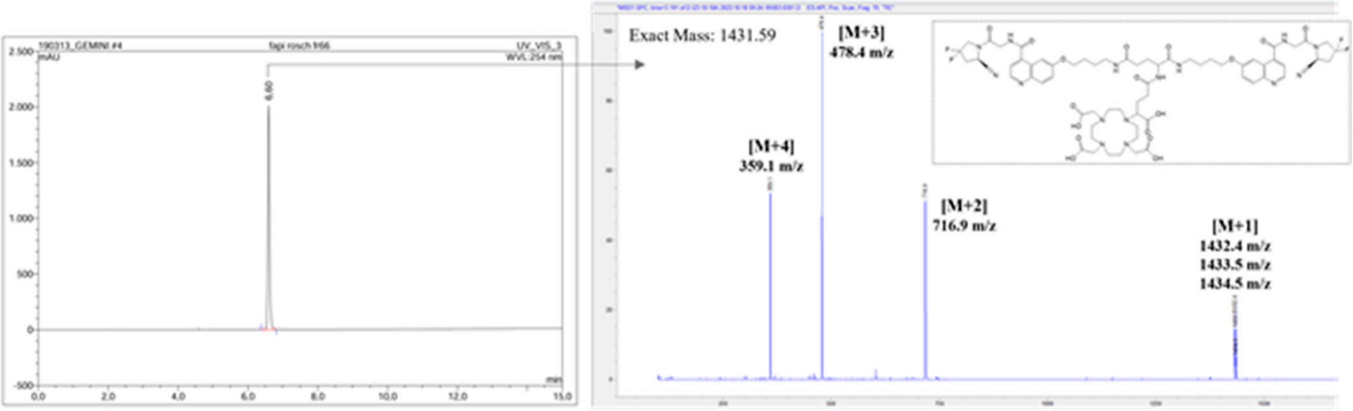

Figure S1. LC-MS and HPLC-MS analyses of the final compound. The pictures show the total ion count (TIC) distribution and the purity of DOTAGA-Glu-(FAPi)$_2$. Analytical HPLC-MS (t_R = 6.60 min), 90% of eluent B (90% MeCN + 10% H_2O + 0.1% TFA) in 15 min: purity = 99%.

a)

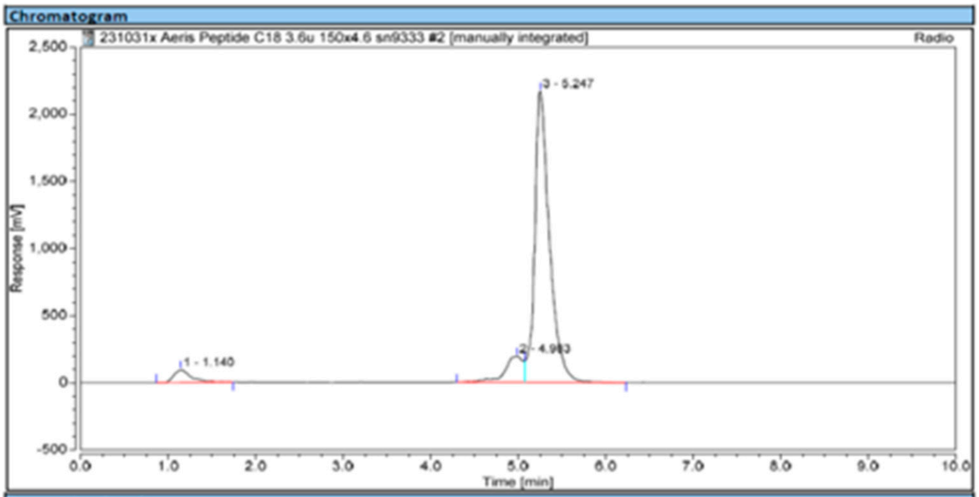

Integration Results

No.	Peak Name	Retention Time min	Area mV*min	Height mV	Relative Area %	Relative Height %	Amount n.a.
1		1.140	21.911	96.734	4.44	3.93	n.a.
2		4.983	47.670	195.688	9.66	7.95	n.a.
3		5.247	423.702	2170.493	85.89	88.13	n.a.
**Total:**			**493.283**	**2462.914**	**100.00**	**100.00**	

b)

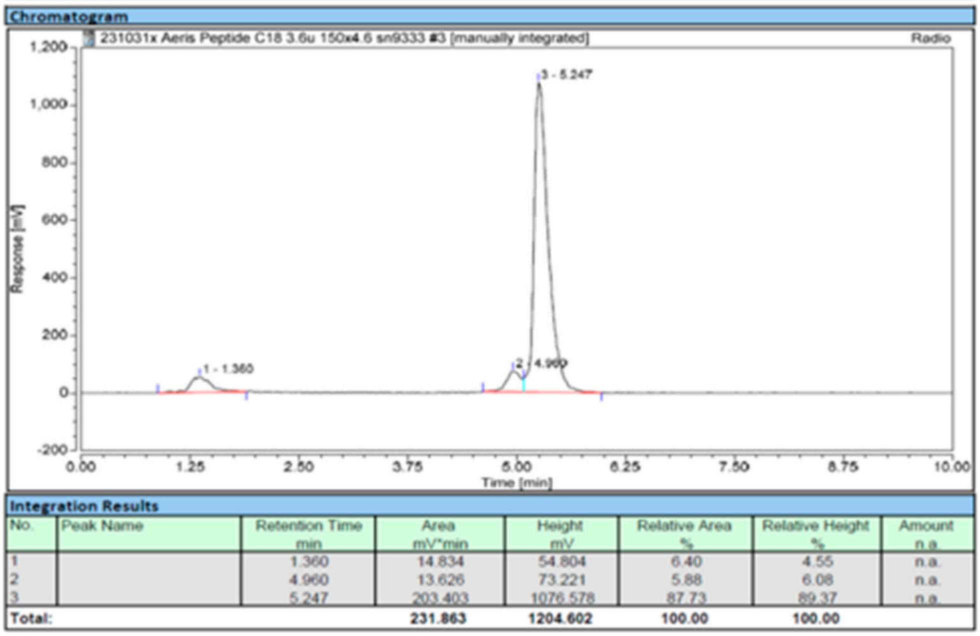

Integration Results

No.	Peak Name	Retention Time min	Area mV*min	Height mV	Relative Area %	Relative Height %	Amount n.a.
1		1.360	14.834	54.804	6.40	4.55	n.a.
2		4.960	13.626	73.221	5.88	6.08	n.a.
3		5.247	203.403	1076.578	87.73	89.37	n.a.
**Total:**			**231.863**	**1204.602**	**100.00**	**100.00**	

Figure S2. Radio-HPLC chromatograms for the preparation of [^{68}Ga]Ga-DOTAGA-Glu-(FAPi)$_2$: (**a**) crude [^{68}Ga]Ga-DOTAGA-Glu-(FAPi)$_2$ in ammonium acetate buffer at the end of heating; (**b**) formulated [^{68}Ga]Ga-DOTAGA-Glu-(FAPi)$_2$. Peak #1 corresponds to unchelated ^{68}Ga, and peak #3 corresponds to [^{68}Ga]Ga-DOTAGA-Glu-(FAPi)$_2$. HPLC conditions: Aeris XB-C18 3.6 μm 150 × 4.6 mm column eluted at 1.5 mL/min with a gradient of ACN in water with 0.1% TFA (*v*/*v*) in both solvents. Gradient protocol: 0–1 min—5% ACN, 1–8 min—increase from 5% ACN to 75% ACN, 8–9 min—75% ACN, 9–9.5 min—decrease to 5% ACN, and 9.5–10 min—5% ACN.

Table S1. Organ distribution in the percentage of injected dose per gram (%ID/g) of [^{68}Ga]Ga-DOTAGA-Glu-(FAPi)$_2$ in the tumor, blood, muscle, kidney, and liver, respectively, at 5, 15, 25, 35, and 45 min post-injection obtained from PET/CT data.

Organ	Mouse	5 min.	15 min.	25 min.	35 min.	45 min.
Tumor	M1	3.52	4.56	4.60	4.68	4.68
	M2	2.48	3.94	4.20	4.34	4.40
	M3	2.54	5.16	5.65	5.95	6.01
	M4	1.66	3.44	3.51	3.52	3.52
	Mean±SEM	2.55±0.38	4.28±0.37	4.49±0.45	4.62±0.51	4.65±0.52
Bladder	M1	4.80	20.10	31.21	35.81	39.77
	M2	4.51	25.42	41.63	47.82	49.32
	M3	3.00	30.39	55.86	69.95	75.38
	M4	3.51	24.10	41.46	47.53	50.79
	Mean±SEM	3.96±0.42	25±2.12	42.54±5.06	50.28±7.13	53.81±7.59
Blood	M1	5.98	4.04	3.66	3.44	3.39
	M2	5.59	4.69	4.16	3.90	3.70
	M3	6.02	4.95	3.86	3.21	2.92
	M4	4.44	5.00	4.62	4.13	3.99
	Mean±SEM	5.51±0.37	4.67±0.22	4.08±0.21	3.67±0.21	3.50±0.23
Muscle	M1	1.00	1.34	1.29	1.28	1.33
	M2	0.74	1.20	1.25	1.37	1.29
	M3	0.65	1.11	1.16	1.24	1.18
	M4	0.43	1.02	1.16	1.06	1.01
	Mean±SEM	0.71±0.12	1.17±0.07	1.22±0.03	1.24±0.07	1.20±0.07
Kidney	M1	7.50	6.09	3.47	2.39	1.99
	M2	7.11	6.56	3.78	2.64	2.17
	M3	6.77	8.67	5.77	4.04	2.73
	M4	5.32	7.01	3.07	2.06	1.74
	Mean±SEM	6.68±0.48	7.08±0.56	4.02±0.60	2.78±0.44	2.16±0.21
Liver	M1	6.93	3.79	2.94	2.67	2.47
	M2	6.06	3.93	3.20	2.93	2.78
	M3	6.15	4.45	3.49	3.06	2.91
	M4	4.19	3.92	3.19	2.85	2.75
	Mean±SEM	5.83±0.58	4.02±0.15	3.21±0.11	2.88±0.08	2.73±0.09

Table S2. Tumor-to-blood ratio (TBR), tumor-to-muscle ratio (TMR), tumor-to-kidney ratio (TKR), and tumor-to-liver ratio (TLR) of [^{68}Ga]Ga-DOTAGA-Glu-(FAPi)$_2$ at 5, 15, 25, 35, and 45 min post-injection obtained from PET/CT data.

Ratio	Mouse	5 min.	15 min.	25 min.	35 min.	45 min.
Tumor-to-blood	M1	0.59	1.13	1.26	1.36	1.38
	M2	0.44	0.84	1.01	1.11	1.19
	M3	0.42	1.04	1.46	1.85	2.06
	M4	0.37	0.69	0.76	0.85	0.88
	Mean±SEM	0.46±0.05	0.92±0.10	1.12±0.15	1.29±0.21	1.38±0.25
Tumor-to-muscle	M1	3.52	3.40	3.57	3.66	3.52
	M2	3.35	3.28	3.36	3.17	3.41
	M3	3.91	4.65	4.87	4.80	5.09
	M4	3.86	3.37	3.03	3.32	3.49
	Mean±SEM	3.66±0.13	3.68±0.32	3.71±0.40	3.74±0.37	3.88±0.41
Tumor-to-kidney	M1	0.47	0.75	1.33	1.96	2.35
	M2	0.35	0.60	1.11	1.64	2.03
	M3	0.38	0.59	0.98	1.47	2.20
	M4	0.31	0.49	1.14	1.71	2.02
	Mean±SEM	0.38±0.03	0.61±0.05	1.14±0.07	1.70±0.10	2.15±0.08
Tumor-to-liver	M1	0.51	1.20	1.56	1.75	1.89
	M2	0.41	1.00	1.31	1.48	1.58
	M3	0.41	1.16	1.62	1.94	2.07
	M4	0.40	0.88	1.10	1.24	1.28
	Mean±SEM	0.43±0.03	1.06±0.07	1.40±0.12	1.60±0.16	1.71±0.17
